# Development and psychometric assessment of the COPD and Asthma Sleep Impact Scale (CASIS)

**DOI:** 10.1186/1477-7525-7-98

**Published:** 2009-12-07

**Authors:** Robin F Pokrzywinski, David M Meads, Stephen P McKenna, G Alistair Glendenning, Dennis A Revicki

**Affiliations:** 1 United BioSource Corporation, Bethesda, MD, USA; 2 Health Outcomes Research, Galen Research, Manchester, UK; 3 Department of Psychology, University of Central Lancashire, Preston, UK; 4 Novartis Research Center, Horsham, UK

## Abstract

**Background:**

Patients with respiratory disease experience disturbed sleep, but there is no widely accepted measure of sleep impairment due to respiratory disease. We developed and evaluated the psychometric performance of a patient-reported measure to assess the impact on sleep due to respiratory disease, the COPD and Asthma Sleep Impact Scale (CASIS).

**Methods:**

Identification of the items forming the CASIS was guided by patient interviews and focus groups. An observational study involving patients from the US and UK was then conducted to assess the psychometric characteristics of the measure.

**Results:**

Qualitative data from 162 patients were used to develop the CASIS (n = 78 COPD; n = 84 asthma). The observational study included 311 patients with COPD and 324 patients with asthma. The final seven items used in the CASIS were identified based on factor and item response theory analyses. Internal consistency was 0.90 (COPD) and 0.92 (asthma), and test-retest reliability was 0.84 (both groups). In the COPD sample, CASIS scores were significantly correlated with the Saint George's Respiratory Questionnaire scores (all p < 0.0001) and differed significantly by patient-reported disease severity, exacerbation status, and overall health status (all p ≤ 0.005). In the asthma sample, CASIS scores were significantly correlated with the Asthma Quality of Life Questionnaire scores (all p < 0.0001) and differed significantly by clinician and patient-reported disease severity, exacerbation status, and overall health status (all p ≤ 0.0005).

**Conclusion:**

The CASIS shows good internal consistency, test-retest reliability, and construct validity and may be useful in helping to understand the impact that COPD and asthma have on sleep outcomes.

## Introduction

Nocturnal symptoms and sleep disturbance are common in both asthma and chronic obstructive pulmonary disease (COPD), particularly with increasing disease severity [1-3]. In patients with COPD, dyspnea is associated with both fatigue and sleep difficulty [3]. Thirty-nine percent of individuals with cough or wheeze in the general population report having problems initiating or maintaining sleep, and this rate increases to 53% in those with both cough and wheeze [4]. In epidemiologic studies, more than 50% of patients with COPD complained of difficulty maintaining and initiating sleep, and 25% complained of excessive daytime sleepiness [5,6]. Patients with asthma also report increased sleep-related disturbances [2]. Elderly patients with asthma reported poorer quality of sleep than elderly patients with no respiratory disease [7].

Information on sleep-related outcomes is important to help understand the impact of disease in patients with asthma or COPD, yet until now these outcomes have been challenging to measure. Although there are several sleep-related measures [8-10], these measures are not specific to respiratory diseases. For example, the Pittsburg Sleep Quality Index [8] covers several relevant aspects of sleep disturbance, but includes other items (e.g., bad dreams, get up to use bathroom) which may not be associated with asthma or COPD. In addition, daily diaries have been used to track sleep difficulty [11], daytime sleepiness and tiredness, difficulty maintaining sleep, early morning awakenings [12], sleep disturbance [13-15], and sleep symptoms [16]. However, each measures only selective aspects of sleep, and it is difficult to compare results between studies. A patient-reported outcome measure targeting sleep impairment in patients with asthma or COPD would complement existing health outcome measures and extend our understanding of the impact of respiratory disease symptoms on sleep impact and quality.

Given the prevalence of sleep problems in patients with asthma or COPD and the absence of respiratory-specific sleep measures, a new measure of sleep problems and impairment was developed, the COPD and Asthma Sleep Impact Scale (CASIS). The CASIS was intended to be brief, patient-centered, and sufficiently comprehensive to capture the sleep problems experienced by patients with respiratory disease.

## Methods

The CASIS was developed in three stages: (1) qualitative research to identify the conceptual framework and items in the measure; (2) cognitive interviewing to ensure patient understanding; and (3) psychometric analysis to evaluate the reliability and validity of the CASIS. Each stage was executed in the US and UK. Relevant IRB and ethics committee approvals were obtained, and all participating patients provided written informed consent. We outline here the methods and results of the first two stages before fully describing the methods for the psychometric analysis.

### Stage 1: Qualitative Research to Identify Conceptual Framework and Item Content

Four focus groups were conducted in the US, two among patients with COPD and two among patients with asthma. One-on-one interviews were conducted in the UK. In total 43 patients with COPD and 55 patients with asthma participated in the qualitative research (Table 1). Content analysis of patient comments was used to identify key concepts and item content relating to nocturnal symptoms (i.e., coughing, difficulty breathing, etc.), trouble falling and remaining asleep, waking during the night, disturbed sleep, feeling tired and not rested when waking up, and worsening respiratory symptoms during the night. The initial 20-item draft CASIS was generated based directly on patient actual statements. Draft questions were reviewed to ensure that they were relevant for US and UK English. A five-level response scale was developed ranging from 1 = none of the time to 5 = all of the time.

**Table 1:** Demographic Characteristics and Health Status for Asthma and COPD Sample

Characteristics	Focus group/interview sample		Cognitive debriefing interview sample		Validation sample	
	
	Asthma (n = 55)	COPD (n = 43)	Asthma (n = 29)	COPD (n = 35)	Asthma (N = 324)	COPD (N = 311)
**Age(years)**						
Mean (SD)	45.2 (17)	66.0 (11)	49.6 (17)	65.2 (9)	48.1 (16)	70.6 (10)
Range	22-82	33-91	18-82	48-80	18-85	45-95

**Male n (%)**	22 (40)	24 (56)	12 (41)	17 (49)	90 (28)	141 (45)

**Years since diagnosis**						
Mean (SD)	20.4 (14)^a^	11.2 (11)	22.3 (16)	11.5 (14)	20.1 (15)	10.5 (11)
Range	1-68	1-50	1-60	1-60	1-71	1-76
Missing, n (%)	1 (2)	1 (2)	1 (3)	3 (9)		

**Participant-reported disease severity, n (%)^b, c^**						
Mild	27 (49)	7 (16)	9 (56)	7 (37)	146 (45)	52 (17)
Moderate	14 (25)	16 (37)	6 (38)	4 (21)	120 (37)	139 (45)
Quite severe	10 (18)	12 (28)	1 (6)	5 (26)	43 (13)	93 (30)
Very severe	3 (5)	5 (12)	0	2 (11)	11 (3)	26 (8)
Missing	1 (2)	3 (7)	0	1 (5)		

**Participant-reported overall health, n (%)^d^**						
Very good	5 (13)	0	2 (13)	0	46 (14)	14 (5)
Good	14 (36)	12 (41)	8 (50)	6 (32)	144 (44)	78 (25)
Fair	15 (38)	6 (21)	4 (25)	7 (37)	104 (32)	137 (44)
Poor	5 (13)	11 (38)	2 (13)	6 (32)	27 (8)	78 (25)

**SGRQ^e ^Mean (SD)**						
Symptom score	---	---	---	---	---	57.7 (22.2)
Activity score						73.3 (23.3)
Impacts score						43.6 (25.0)
Total score						54.7 (21.7)

**AQLQ^f ^Mean (SD)**						
Symptoms	---	---	---	---	4.7 (1.4)	---
Activity limitation					5.0 (1.4)	
Emotional function					4.9 (1.6)	
Environmental stimuli					4.6 (1.6)	
Overall					4.8 (1.3)	

^a ^3 asthma participants excluded from mean calculation were diagnosed in early childhood/infancy.

^b ^Clinician-reported for focus group asthma and COPD samples (US only)

^c ^Cognitive debriefing interview sample collected in UK only: asthma n = 16; COPD n = 19

^d ^Focus group/interview samples collected in UK only: asthma n = 39; COPD n = 29. Cognitive debriefing interview samples collected in UK only: asthma n = 16; COPD n = 19

^e ^Scores range from 0 to 100 with higher scores reflecting more impairment. COPD participants only.

^f ^Scores range from 1 to 7 with higher scores reflecting more favorable health status. Asthma participants only.

### Stage 2: Cognitive Debriefing Interviews

Individual interviews in the US and UK were conducted to assess respondent comprehension of the 20-item draft CASIS. Thirty-five patients with COPD and 29 patients with asthma were interviewed (Table 1). Based on this qualitative research, several items were modified, and five items that were not well-understood or redundant were removed, resulting in a 15-item CASIS. The response options were revised to range from 1 = never to 5 = very often. This revised 15-item CASIS was then used in the psychometric evaluation study.

### Stage 3: Psychometric Evaluation Study

#### Study Design

An observational study was designed and conducted among patients with either COPD or asthma from the US and UK with the objective of evaluating item performance and psychometric properties of the CASIS. The CASIS was completed along with other selected patient-reported outcomes (PRO) instruments. Inclusion criteria for the study were: (1) a clinical diagnosis of COPD or asthma; (2) age 18 years or older; and (3) willingness to provide consent to participate. Patients were excluded if they had both asthma and COPD; had a severe comorbid chronic medical conditions perceived to be unstable by the principal investigator (e.g., congestive heart failure, diabetes, schizophrenia, depression, etc.); or, in the investigator's judgment, had cognitive impairments or other conditions that would make study participation difficult. All patients completed a baseline study visit, and a subset of US patients completed the second visit within two weeks (10-14 days) of the baseline visit. All UK patients completed a mail survey at baseline, with a follow-up mail survey two weeks later.

#### Health Outcome Measures

All patients completed a demographic form at baseline and a change in health status form at the follow-up visit. All patients rated their disease severity (mild, moderate, severe) and general health status (poor, fair, good, very good). In the US, clinicians rated disease severity (mild, moderate, severe) and exacerbations during the previous month. At baseline, the COPD patients reported whether they used oxygen and the number of "bad days" due to their disease during the previous week.

The CASIS was administered at two time points. Patients with COPD completed the St. George's Respiratory Questionnaire (SGRQ) [17] and the Living with Chronic Obstructive Pulmonary Disease Questionnaire (LCOPD; McKenna SP, Meads DM, Doward LC, Pokrzywinski RF, Revicki DA, Hunter CJ, Glendenning GA: Development and validation of the Living with Chronic Obstructive Pulmonary Disease [LCOPD] Questionnaire, submitted) during their baseline visit or survey. Patients with asthma completed the Asthma Quality of Life Questionnaire (AQLQ) [18], and the Asthma Life Impact Scale (ALIS) (Meads DM, McKenna SP, Doward LC, Pokrzywinski RF, Revicki DA, Hunter CJ, Glendenning GA: Development and validation of the Asthma Life Impact Scale [ALIS], submitted) during the baseline visit or survey.

##### COPD and Asthma Sleep Impact Scale

The 15-item CASIS incorporated items on sleep impairment associated with respiratory disease and breathing problems. The response options ranged from 1 = never to 5 = very often, and several items are reverse-scored. The item scores were summed together to arrive at a total raw score. CASIS raw scores were linearly transformed to a 0-100 total scale score. Recall time period for the measure was the previous week, with higher scores indicating greater sleep impairment.

##### St. George's Respiratory Questionnaire

The SGRQ is a self-administered instrument that assesses the health status of patients with COPD or other chronic airflow limitations [17]. The SGRQ is commonly used in COPD research studies and has evidence supporting reliability, validity, and responsiveness [17].

##### Living with Chronic Obstructive Pulmonary Disease Questionnaire

The LCOPD is a measure developed to assess the daily impact of living with COPD (McKenna SP, Meads DM, Doward LC, Pokrzywinski RF, Revicki DA, Hunter CJ, Glendenning GA: Development and validation of the Living with Chronic Obstructive Pulmonary Disease [LCOPD] Questionnaire, submitted). For the LCOPD, higher scores represent greater impairment to quality of life.

##### Asthma Quality of Life Questionnaire

The standardized AQLQ is a self-administered asthma-specific health-related quality of life (HRQL) instrument that is widely used to assess outcomes in asthma [18]. The AQLQ has good evidence supporting reliability, validity, and responsiveness and is widely used for measuring HRQL in adults with asthma [18].

##### Asthma Life Impact Scale

The ALIS is a measure developed to assess the daily impact on patients of living with asthma (Meads DM, McKenna SP, Doward LC, Pokrzywinski RF, Revicki DA, Hunter CJ, Glendenning GA: Development and validation of the Asthma Life Impact Scale [ALIS], submitted). For the ALIS, higher scores represent a greater impairment of asthma on the quality of life.

#### Psychometric Analyses

Psychometric analyses and item response theory (IRT) analysis examined item performance [19,20]. After the final CASIS items were determined then the reliability and validity of the measure was evaluated [21]. All data analyses were performed using SAS statistical software version 9.1 (Cary, NC) and Multilog [22].

#### Item Performance and Reduction

Item analyses used to evaluate the CASIS included item-to-item correlations using Pearson's correlations, factor analyses, and IRT analyses. The factor analyses were used to examine the unidimensionality of the CASIS items (i.e., measures a single construct), and the IRT analysis evaluated item performance. Differential item functioning (DIF) was conducted to evaluate whether there were differences in item responses by disease or country.

#### Reliability

Internal consistency reliability was measured using Cronbach's alpha [23]. Intraclass correlations (ICC), Pearson's correlations, and change scores between the baseline and follow-up were used to evaluate test-retest reliability and stability of the CASIS scores. The ICC was estimated using a fixed-effects analysis of variance (ANOVA) model [24]. ICCs greater than 0.70 are acceptable for group comparisons [24].

#### Construct and Known Groups Validity

Spearman correlations were used to evaluate construct validity of the CASIS through correlations between baseline CASIS scores and overall health status, SGRQ, and LCOPD scores in the COPD sample and between CASIS scores and overall health status, ALIS, and AQLQ scores in the asthma sample [25]. Moderate correlations between the CASIS and the disease-specific PRO measures were expected (i.e., r = 0.30-0.60).

To examine known groups validity by disease severity, participants were stratified according to the clinician-rated and patient-rated disease severity (i.e., mild, moderate, severe). Mean CASIS scores were compared by severity, overall health rating (i.e., poor, fair, good, very good), and exacerbation status using ANOVA.

## Results

Six hundred and thirty-five patients were enrolled in the observational study in the US (n = 333) and UK (n = 302). Of the participants, 311 were diagnosed with COPD and 324 with asthma (Table 1).

### Item Performance and Reduction

There were minimal missing data for the items (<2.5%), and the entire range of response options was used. There were minimal ceiling effects and four items with floor effects (lowest response > 30%). Nine items were highly correlated (r ≥ 0.75) with other items indicating item redundancy.

A 1-factor exploratory factor analysis was completed for the asthma and COPD samples separately. Inspection of the factor loadings indicated that they were similar across the COPD and asthma samples (data not shown).

For the 15 items, IRT analyses were conducted separately using the combined sample, COPD sample, and asthma sample. For the combined sample, slope coefficients for the IRT grade response model ranged from 4.06 to 1.65 (data not shown). The slope coefficients reflect the strength of the association between the individual items and the underlying contract, in this case sleep impairment. Based on the IRT analyses, CASIS item responses represented good coverage of the concept sleep impairment based on a review of the category threshold parameters. The IRT analyses of the COPD and the asthma samples were similar.

Based on the item analyses, we removed eight items. Four items were removed due to redundancy and four based on floor effects and redundancy. The psychometric characteristics were determined for the final seven-item CASIS (see Additional File 1).

A confirmatory factor analysis was performed using the combined sample restricted to the seven items selected for the final CASIS. In the COPD and asthma samples, the single factor solution explained 58% (in COPD) to 62% (in asthma) of the variance in the items. Factor loadings were comparable across the two samples (data not shown) confirming the unidimensional structure of the CASIS items across disease groups. DIF analyses indicated no significant differences in the pattern of responses to CASIS items by disease or country (data not shown).

### Reliability

Internal consistency reliability for the CASIS was 0.91, 0.90, and 0.92 for the combined, COPD, and asthma samples, respectively. Test-retest reliability was assessed in the sub-sample of participants who reported no change in their health between the initial and follow-up assessments (COPD: n = 112; Asthma: n = 61). The ICCs were 0.84 in both groups (Table 2).

**Table 2:** Test-retest Reliability for the CASIS Scores in Stable Patients with Asthma or COPD

CASIS	N	Visit 1 Mean (SD)	Visit 2 Mean (SD)	Difference Score	P-Value^1^	Pearson's r^2^	ICC^3^
COPD Participants	112	47.1 (24.0)	45.7 (23.5)	-1.5	0.2443	0.85	0.84

Asthma Participants	61	44.8 (27.2)	42.0 (26.6)	-2.7	0.1703	0.84	0.84

^1 ^Paired t-tests comparing responses at baseline and follow-up

^2 ^Pearson product moment correlations

^3 ^Intraclass correlation coefficient

### Validity

For the COPD sample, there were significant correlations between CASIS scores and number of bad days (r = 0.61, p < 0.0001), overall health status (r = 0.50, p < 0.0001), LCOPD (r = 0.58, p < 0.0001), the SGRQ total score (r = 0.68, p < 0.0001) and SGRQ domain scores (r = 0.53 to 0.65, all p < 0.0001) (Table 3). For the asthma sample, significant correlations were found between CASIS scores and overall health status (r = 0.48, p < 0.0001), ALIS (r = 0.59, p < 0.0001), and AQLQ overall score (r = -0.72, p < 0.0001) and domain scores (r = -0.49 to -0.75, p < 0.0001) (Table 4).

**Table 3:** Correlations Between CASIS and Other PRO Measures--COPD Sample: Baseline Data

PRO Measure	Spearman Correlation
Number of bad days^1^	0.61
Overall Health Status	0.50
LCOPD	0.58
SGRQ--Symptoms	0.57
SGRQ--Activities	0.53
SGRQ--Impact	0.65
SGRQ--Total Score	0.68

^1 ^Number of bad days in the past week

All correlations significant at p < 0.0001

**Table 4:** Correlations Between CASIS and Other PRO Measures--Asthma Sample: Baseline Data

PRO Measure	Spearman's Correlation
Overall Health Status	0.48
ALIS	0.59
AQLQ--Symptoms	-0.75
AQLQ--Activity Limitation	-0.68
AQLQ--Emotional Function	-0.59
AQLQ--Environmental Stimuli	-0.49
AQLQ--Overall Score	-0.72

All correlations significant at p < 0.0001

**Figure 1:**
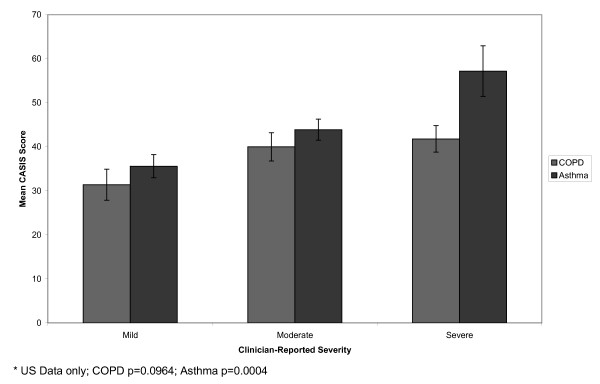
Mean CASIS Scores by Clinician-reported Severity*.

**Figure 2:**
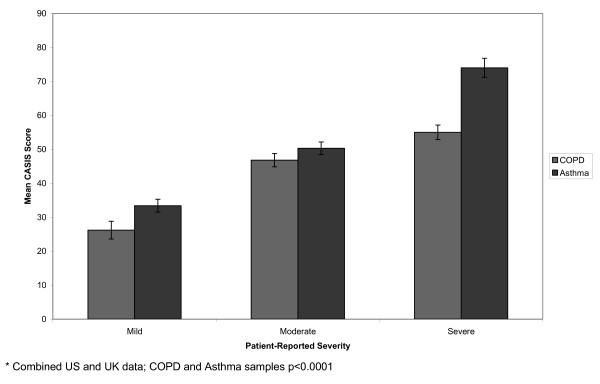
Mean CASIS Scores by Patient-reported Severity*.

**Figure 3:**
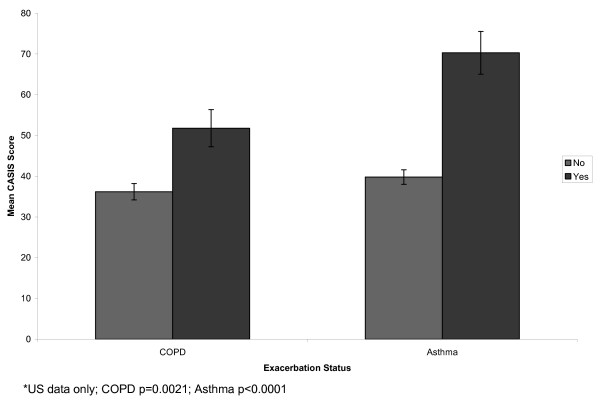
Mean CASIS Scores by Exacerbation Status*.

**Figure 4:**
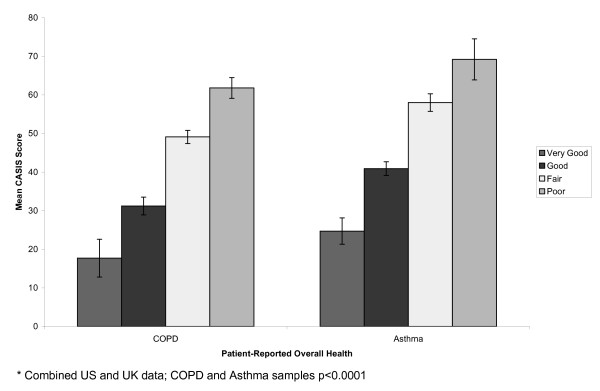
Patient-reported Overall Health*.

Participants were stratified by clinician-reported severity (Figure 1), patient-reported severity (Figure 2), exacerbation status (Figure 3), and overall health status (Figure 4). In the COPD sample, mean CASIS scores differed significantly by patient-reported severity (p < 0.0001), exacerbation status (p = 0.0021), and overall health status (p < 0.0001), but not clinician-rated severity (p = 0.0964). Based on clinician-rated severity, there were clear differences between the mild and moderate or severe groups, but not between the moderate and severe COPD groups (Figure 1). Mean CASIS scores were significantly more impaired in those patients with COPD receiving oxygen (mean = 51.4; SD = 23.1 versus mean = 43.3; SD = 24.7, p = 0.004). In the asthma sample, mean CASIS scores differed significantly by clinician-rated severity (p = 0.0004), patient-reported severity (p < 0.0001), exacerbation status (p < 0.0001), and overall health status (p < 0.0001).

## Discussion

Focus groups and patient interviews generated the item content for the CASIS, and information from cognitive interviews was used to refine the CASIS. Psychometric analyses based on a sample of 635 patients with either asthma or COPD were used to finalize the item content and to evaluate reliability and validity.

Based on the factor analyses and the IRT analyses, there was evidence to show that the scale measures one construct--in this case, sleep impairment due to respiratory disease. Analyses also demonstrated that the CASIS items performs in a similar way in the US and UK and in patients with COPD or asthma. This finding may be justified because although there are clinical differences in the disease experience between asthma and COPD, the CASIS was designed to assess the impact of this disease experience on sleep related outcomes.

The CASIS demonstrated excellent internal consistency reliability (> 0.90) and very good test-retest reliability (> 0.80). These reliability statistics exceed commonly accepted criteria for group data and therefore indicate that the CASIS has good reliability [21,26].

Construct validity of the CASIS scores was demonstrated through evaluation of correlations with other relevant PRO measures in the COPD and asthma samples. As expected, moderate to large significant correlations between CASIS scores and the PRO measures were observed. Greater sleep impairment was associated with greater levels of impaired activities, symptoms, and poorer quality of life for the COPD sample and greater levels of impaired activities, symptoms, social functioning, and poorer quality of life scores for the asthma sample. The construct validity results suggest COPD and asthma symptoms impact sleep and are related to impaired health status and HRQL.

CASIS scores significantly differed between levels of self-reported severity, self-reported overall health, and exacerbation status for COPD patients. In addition, CASIS scores were significantly more impaired in patients with COPD receiving oxygen compared with those not receiving oxygen. The CASIS was not able to significantly distinguish between clinician-reported severity levels for patients with COPD. However, there was a clear differentiation between the mild and moderate to severe COPD groups, but not between the moderate and severe groups. A possible reason could be the lack of lung volume cut points for the clinician ratings, and variability in clinician definitions of moderate and severe COPD. In addition, patient-rated and clinician-rated severity differed and this may be the source of non-significant differences in the COPD group. We found agreement between clinician-rated severity and patient-rated severity with a kappa statistic of 0.39 in the COPD sample and 0.45 for the asthma sample. In the asthma group, the CASIS was able to significantly distinguish between clinician-rated and patient-rated severity, overall health, and exacerbation status for patients with asthma. The ability of the CASIS to differentiate by disease severity and overall health status suggest that the CASIS may be sensitive to change in clinical severity.

The CASIS uses a one-week recall period, and this recall period was consistent with the preferences of the patients in our study. A one-week recall period allows for a broader experience to be captured given the varied impact of respiratory symptoms on sleep problems. This recall period is also consistent with the recently developed symptom and functional outcomes, including sleep/wake function, for the Patient Reported Outcome Measurement Information System [27].

Several study limitations should be considered when interpreting these results. First, the generalizability of the psychometric evaluation to the larger COPD and asthma population may be limited given that we excluded patients with unstable chronic medical conditions. Second, there is no evidence as to the responsiveness of the CASIS to changes in clinical status and this should be evaluated in future research. Previous research, however, suggests that PRO instruments that demonstrate good known groups validity also tend to demonstrate responsiveness [26]. The CASIS was highly successful in discriminating based on exacerbation status, patient-reported severity, patient-reported overall health, and clinician-reported severity (asthma group only). Clinician-reported severity, for the COPD sample, did not reach statistical significance so future research is needed to address this potential limitation. Third, for some of the known group comparisons, there were relatively small samples for some groups (i.e., exacerbations). Finally, pulmonary function or polysomnography measures were not collected, and future research is needed to examine the relationship between these clinical measures and the CASIS.

The CASIS was developed to measure sleep impairment associated with respiratory disease, specifically asthma and COPD. This new instrument provides a respiratory disease-specific measure of sleep impairment that can complement more generic sleep problem measures. The CASIS differs from more generic sleep measures because it was developed by focusing on the experience of patients with either asthma or COPD, excludes content targeted to generic sleep deprivation (e.g., bad dreams, get up to use bathroom, etc), and includes respiratory specific sleep items (e.g., wake up at night with breathing problems, etc.). The measure was developed simultaneously in the US and the UK. The methods used to systematically develop the CASIS contribute to the strength of this new PRO measure. Based on the current study, there is good evidence supporting the internal consistency reliability, test-retest reliability, and concurrent and known groups validity in patients with either asthma or COPD. The CASIS may prove to be a useful measure of sleep impairment due to respiratory disease and help to understand the impact that COPD and asthma have on outcomes related to sleep.

## Abbreviations

ALIS: Asthma Life Impact Scale; ANOVA: Analysis of variance; AQLQ: Asthma Quality of Life Questionnaire; CASIS: COPD and Asthma Sleep Impact Scale; COPD: Chronic obstructive pulmonary disease; DIF: Differential item functioning; HRQL: Health-related quality of life; ICC: Intraclass correlation; IRT: Item response theory; LCOPD: Living with Chronic Obstructive Pulmonary Disease Questionnaire; PRO: Patient-reported outcome; SGRQ: St. George's Respiratory Questionnaire.

## Competing interests

This research was supported by funding from Novartis. DAR, RFP, DMM, and SPM receive research funding from Novartis. GAG is an employee of Novartis.

## Authors' contributions

RFP managed the US portion of the research study, including developing protocols; site coordination; data collection; data oversight; and reporting of data. DMM managed the UK portion of the research study, including developing protocols; site coordination; data collection; data oversight; and reporting of data. SPM served as Principal Investigator and Research Leader for all UK activities, including study design and direction, interpretation of data; and reporting of data. GAG developed the conceptual need for such work in the area of health outcomes research, contributed to the conceptual design and critical content of the research study. DAR served as Principal Investigator and Research Leader for all US activities, including study design and direction, interpretation of data; and reporting of data.

All authors have read and approved the final manuscript.

## Supplementary Material

Additional file 1 **COPD and Asthma Sleep Impact Scale**. This is the COPD and Asthma Sleep Impact Scale referred to in the manuscript. Click here for file
