# Supplementary material for: Development and psychometric assessment of the COPD and Asthma Sleep Impact Scale (CASIS)
Source: Health Qual Life Outcomes. 2009 Dec 7;7:98. doi: 10.1186/1477-7525-7-98 (PMC2794842; doi:10.1186/1477-7525-7-98)
Supplement: Additional file 1 — COPD and Asthma Sleep Impact Scale. This is the COPD and Asthma Sleep Impact Scale referred to in the manuscript. [file 1477-7525-7-98-S1.DOC]

# Additional file 1: COPD and Asthma Sleep Impact Scale

**Instructions:** For each question below, please check 🗹 the response that best represents how you feel. When answering the questions, please think about the impact of breathing problems/COPD/asthma on your sleep during the past week, even if the past week was unusually good or unusually bad.

| **During the past week, how often did you:** | **Never** | **Rarely** | **Sometimes** | **Often** | **Very Often** |
| --- | --- | --- | --- | --- | --- |
| 1. have a bad night’s sleep? | 🞎 | 🞎 | 🞎 | 🞎 | 🞎 |
| 1. have problems staying awake during the day? | 🞎 | 🞎 | 🞎 | 🞎 | 🞎 |
| 1. have trouble falling asleep? | 🞎 | 🞎 | 🞎 | 🞎 | 🞎 |
| 1. wake up at night with breathing problems (shortness of breath, coughing, chest tightness, etc.)? | 🞎 | 🞎 | 🞎 | 🞎 | 🞎 |
| 1. wake up during the night and have trouble falling back asleep? | 🞎 | 🞎 | 🞎 | 🞎 | 🞎 |

Please think about the impact of your breathing problems during the past week, and check 🗹 the response that best represents how you feel.

| **During the past week, how often did you:** | **Never** | **Rarely** | **Sometimes** | **Often** | **Very Often** |
| --- | --- | --- | --- | --- | --- |
| 1. have a good night’s sleep? | 🞎 | 🞎 | 🞎 | 🞎 | 🞎 |
| 1. wake up feeling rested? | 🞎 | 🞎 | 🞎 | 🞎 | 🞎 |
